# Amphiphilic Compounds Assemble into Membranous Vesicles in Hydrothermal Hot Spring Water but Not in Seawater

**DOI:** 10.3390/life8020011

**Published:** 2018-05-10

**Authors:** Daniel Milshteyn, Bruce Damer, Jeff Havig, David Deamer

**Affiliations:** 1 Department of Biomolecular Engineering, University of California Santa, Cruz, CA 95064, USA; dmilshte@ucsc.edu (D.M.); bdamer@digitalspace.com (B.D.); 2 Department of Earth Sciences, University of Minnesota Minneapolis, Minneapolis, MN 55455, USA; jeffhavig@gmail.com

**Keywords:** self-assembly, lipid vesicles, hydrothermal environments

## Abstract

There is a general assumption that amphiphilic compounds, such as fatty acids, readily form membranous vesicles when dispersed in aqueous phases. However, from earlier studies, it is known that vesicle stability depends strongly on pH, temperature, chain length, ionic concentration and the presence or absence of divalent cations. To test how robust simple amphiphilic compounds are in terms of their ability to assemble into stable vesicles, we chose to study 10- and 12-carbon monocarboxylic acids and a mixture of the latter with its monoglyceride. These were dispersed in hydrothermal water samples drawn directly from hot springs in Yellowstone National Park at two pH ranges, and the results were compared with sea water under the same conditions. We found that the pure acids could form membranous vesicles in hydrothermal pool water, but that a mixture of dodecanoic acid and glycerol monododecanoate was less temperature-sensitive and assembled into relatively stable membranes at both acidic and alkaline pH ranges. Furthermore, the vesicles were able to encapsulate nucleic acids and pyranine, a fluorescent anionic dye. None of the amphiphiles that were tested formed stable vesicles in sea water because the high ionic concentrations disrupted membrane stability.

## 1. Introduction

At some point along the pathway to the origin of life, assembly of microscopic compartments containing functional systems of polymers was an essential step [1,2]. Multiple laboratory methods have been devised to prepare vesicles composed of phospholipids—often referred to as liposomes—because they have pharmaceutical value as drug delivery agents, but these methods are highly technical and unsuitable for natural conditions. There are several problems to overcome if polymers were to be encapsulated in the prebiotic environment. We first note that there are two aqueous phases in which life may have emerged. Because most of the Earth’s water today is in the ocean, it is generally assumed that life began in the sea, and hydrothermal vents have been proposed as a possible site [3,4]. However, a reasonable assumption is that volcanic land masses emerged above sea level 4 Gya [5] with hydrothermal hot springs and ponds produced by precipitation distilled from the salty ocean. In contrast to the diluting effect of oceans, water bodies on land would collect and concentrate organic compounds associated with meteoritic infall, providing a source of monomers to be incorporated into polymers such as RNA during the period of life’s emergence [6]. An alternative, then, is that life began in fresh water and later adapted to seawater [2,6,7].

Laboratory simulations of prebiotic conditions typically use pure water, sometimes buffered at specific pH ranges. However, the NaCl concentration in seawater is close to 0.6 M, MgCl_2_ is 54 mM and CaCl_2_ is 10 mM, and these are far beyond the typical concentrations of ions in cytoplasm, which typically are ~100 mM KCl, 5 mM Mg^++^ and less than micromolar concentrations of Ca^++^. As will be presented in detail later, the composition and concentration of ions in hydrothermal pools associated with volcanic land masses more closely resembles that of the cytoplasm [8]. Lipid vesicles prepared in these conditions are usually composed of phospholipids, such as phosphatidylcholine, but it is implausible that phospholipids would be available in the prebiotic conditions because their synthesis today depends on metabolism. Amphiphiles, such as fatty acids and their monoglycerides, are more plausible choices, but the pH and ionic composition of an aqueous phase strongly affects the ability of amphiphilic compounds to assemble into membranes [9,10,11,12,13].

The actual prebiotic conditions in which life originated were likely to be very different from those of laboratory simulations. For this reason, we decided to investigate the properties of membranous vesicles composed of amphiphilic compounds in water samples drawn from hydrothermal springs and in seawater. The following questions were addressed:

1. How does the ionic composition of hydrothermal hot spring water compare with seawater?
2. Can model amphiphilic compounds assemble into stable membranes in hot spring water?
3. Can model amphiphilic compounds form vesicles in seawater?
4. Can membranes assembled from model amphiphilic compounds provide permeability barriers to ionic solutes?
5. Can nucleic acids be encapsulated in vesicles assembled from model amphiphilic compounds?

## 2. Materials and Methods

### 2.1. Amphiphilic Compounds and Water Sampling

Decanoic acid, dodecanoic acid and glycerol monolaurate were purchased from Aldrich and used without further purification.

**Figure 1:**
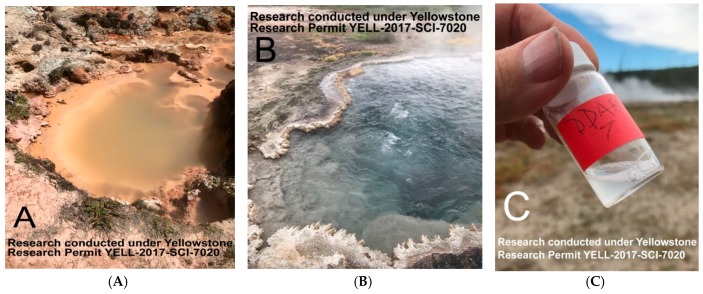
Acidic clay-lined acid pool (**A**), silica-rich alkaline Bison Pool (**B**) and sample vial (**C**) with Bison Pool water added and shaken to disperse the amphiphilic compounds.

Water samples were taken from an acidic pool in the Midway Geyser Basin area (Figure 1A, pH 3.3, 53 °C) and the slightly alkaline Bison Pool in the Lower Geyser Basin area (Figure 1B, pH 7.9, 68 °C). Samples for determining anions and cations were filtered through 0.2 µm polyethersulphone syringe filters (VWR International, Radnor, PA, USA) into 15 mL centrifuge tubes, and stored at 4 °C until analysis. Anion and cation concentrations were determined with instruments in the Analytical Geochemistry Lab in the Department of Earth Sciences, University of Minnesota. Anions (chloride, fluoride and sulfate) were determined via a Thermo Scientific Dionex ICS 5000+ ion chromatography system (Thermo Fisher Scientific, Waltham, MA, USA). Cations (sodium, calcium, magnesium and potassium) were determined via a Thermo Scientific iCAP 6000 Series induced coupled plasma optical emission spectrometer (Thermo Fisher Scientific, Waltham, MA, USA).

Dissolved organic carbon (DOC) samples were filtered as described above into a 50 mL centrifuge tube, flash frozen on dry ice in the field and kept in the dark at or below −20 °C until analysis. DOC was determined at the Stable Isotope Facility (University of California, Davis) via an O.I. Analytical Model 1030 TOC Analyzer (O.I. Analytical, College Station, TX, USA) interfaced to a PDZ Europa 20-20 isotope ratio mass spectrometer (Sercon Ltd., Cheshire, UK) utilizing a GD-100 Gas Trap Interface (Graden Instruments, Oakville, Ontario, Canada).

To investigate self-assembly of membranous vesicles in the field, 3 mL hydrothermal water was added directly to dry powders of decanoic acid and dodecanoic acid with and without added monoglycerides in a 1:1 mole ratio. The weights of the powders were calculated to produce 10 mM amphiphiles, a concentration which preliminary experiments showed to be appropriate for microscopic examination. The samples were shaken by hand to disperse the amphiphiles at the temperature of the source water (Figure 1C), then returned to the laboratory for analysis.

For comparison, a liter of seawater was taken from clear water off the coast of Santa Cruz, CA, then filtered through a 0.22 micrometer Millipore filter to remove particulate matter and microorganisms.

### 2.2. Microscopy

Samples were heated in a 90 °C water bath for 1 min and shaken briefly by hand (~5 s) to disperse the amphiphiles. Aliquots (20 µL) were transferred to a glass slide heated on a laboratory hot plate to 85 °C followed by addition of a cover slip. In some experiments, the slides were examined immediately at 400× magnification. In other experiments designed to monitor whether encapsulation occurred, the sample was allowed to dry ~1 min. After dehydrating samples on a hot plate, the slides were removed, kept warm until the addition of 85 °C water in the rehydration phase followed by a cover slip. The slides slowly cooled after the addition of water, and evaporation of water from beneath the cover slip was minimal. Phase and fluorescence micrographs were taken within 2–3 min while the amphiphiles remained in a fluid state.

### 2.3. Encapsulation of Dye

In order to demonstrate that the vesicles were membrane-bounded compartments capable of maintaining concentration gradients, pyranine dye (8-hydroxypyrene-1,3,6-trisulfonate) was added to 1 mL of each amphiphile dispersion to make a 1.0 mM solution. The dispersions were heated to a temperature above the melting point of the amphiphiles and were then put through a Sephadex G-50 column to separate vesicles from dye. The amount of encapsulated dye was measured by fluorescence (450 nm excitation, 508 nm emission), and vesicles were viewed using 430 nm to excite pyranine fluorescence.

### 2.4. Encapsulation of Nucleic Acids

Preliminary experiments showed that a 1:1 mole ratio mixture of dodecanoic acid (lauric acid) with its monoglyceride formed stable membranes in acidic and alkaline hydrothermal water. Therefore, we focused on this mixture, abbreviated LA–GML (lauric acid–glycerol monolaurate) to investigate encapsulation. To test encapsulation of nucleic acids, LA–GML was mixed in a 2:1 ratio by weight with either Torula yeast ribosomal RNA (CHEM-IMPEX Int’l Inc. Wood Dale, IL) or with lambda phage DNA. The mixture (20 µL) was dried on a glass slide at 85 °C, then rehydrated with 20 µL of water to simulate a wet-dry-wet cycle occurring in an evaporating hydrothermal pool. A fluorescent dye (acridine orange, 0.1 mg/mL) was present during drying to stain the nucleic acids.

To make a quantitative measurement of nucleic acid encapsulation, 10 mM LA–GML was mixed in a 6:1 mass ratio by weight with the yeast RNA. The mixture (800 µL) was dried as 100 µL aliquots on glass slides at 85 °C, then rehydrated with 400 µL of the pH 7.9 Bison Pool water that was used to make the original amphiphilic mixture. After rehydration, bovine pancreatic ribonuclease (10 μg) was added to the solution and incubated at room temperature for 25 min. The ribonuclease was added in order to degrade free RNA into shorter chains that could be separated by gel permeation chromatography, while encapsulated RNA was protected from hydrolysis. After incubation, the mixture was put through a Sephadex G-50 column to separate vesicles with encapsulated RNA from the hydrolyzed RNA products. The amount of encapsulated RNA in the vesicle fraction was measured using a NanoDrop instrument set to the RNA mode in which the absorbance 260 and 280 nm is used to calculate the amount present. This amount was compared to the total RNA that was originally added and expressed as percent encapsulated. A control was also run in which the RNA was added to the lipid vesicles without being dried, then hydrolyzed with RNase before being put through the Sephadex column. There was no detectable absorbance at 260 nm, which confirmed that only the encapsulated RNA was measured.

## 3. Results

The most significant chemical and physical properties of the two hydrothermal water samples related to this study are the pH, ionic composition and dissolved organic carbon (Table 1). The pH of Bison Pool sample was slightly alkaline (pH 7.9), while the Midway Geyser Basin pool was acidic (pH 3.3). Both were at an elevated temperature and the dissolved organic carbon was very dilute, in the micromolar range.

### 3.1. Decanoic and Dodecanoic Acid

**Figure 2:**
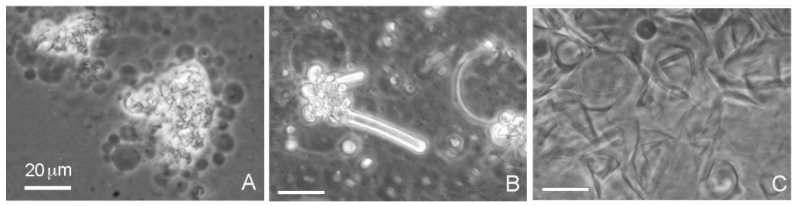
Decanoic acid (10 carbons) formed droplets at an acidic pH range (Geyser Basin sample) but partially crystallized at the alkaline pH range (Bison Pool sample) even above the transition temperature of 31 °C. All bars show 20 micrometer scales.

When decanoic acid was dispersed in acidic water samples at pH 3.3, it clumped into microscopic aggregates, but membranous structures could be observed emerging from the aggregates in the form of vesicles (Figure 2A). Multilamellar myelin figures were also observed (Figure 2B). In the Bison Pool water (pH 7.9) flattened semicrystalline structures dominated and vesicles were less prominent (Figure 2C).

**Figure 3:**
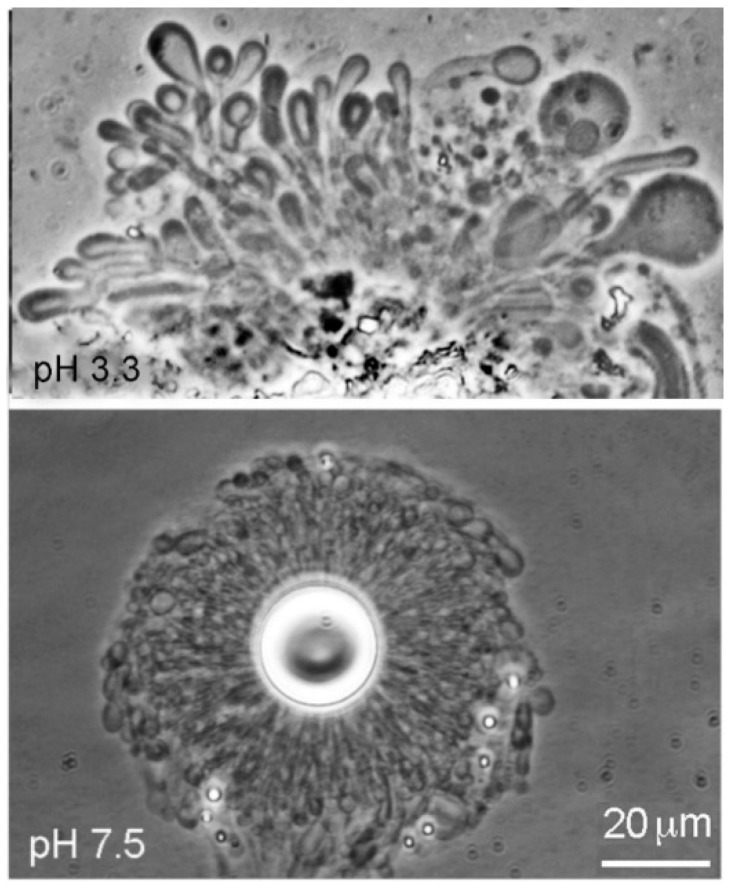
Dodecanoic acid at pH 3.3 (Geyser Basin sample) and pH 7.9 (Bison Pool sample). At both pH ranges the vesicles emerged from melted droplets of the acids.

Dodecanoic acid (12-carbon lauric acid) formed membranous vesicles at pH 3.3 and pH 7.9 (Figure 3). In both cases, the vesicles emerged from a melted droplet of the acid.

### 3.2. Mixtures with Monoglycerides

**Figure 4:**
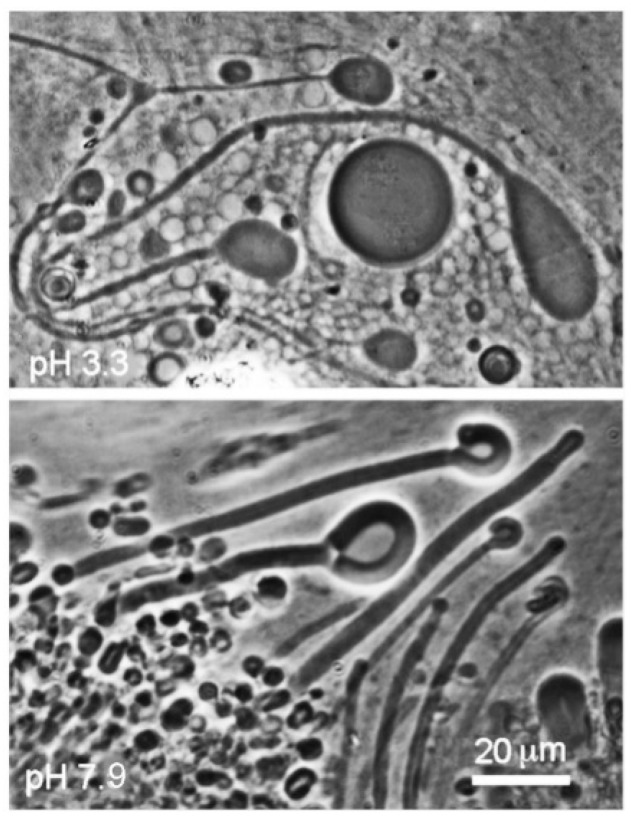
Dodecanoic acid (12 carbons), mixed in a 1:1 mole ratio with its monoglyceride, formed stable membranes at both acidic (Geyser Basin) and alkaline (Bison Pool) pH ranges.

As reported in earlier studies [9,10], mixing monoglycerides with fatty acids markedly enhances the robustness of membranes that form. The results reported here confirm that a 1:1 mole ratio mixture of dodecanoic acid with its monoglyceride (LA–GML) formed stable membranes at both acidic and alkaline pH ranges of hydrothermal water (Figure 4). The mixture also had an enhanced tendency to maintain membranous structures below the transition temperature of lauric acid rather than crystallizing.

### 3.3. Encapsulation of Solutes

**Figure 5:**
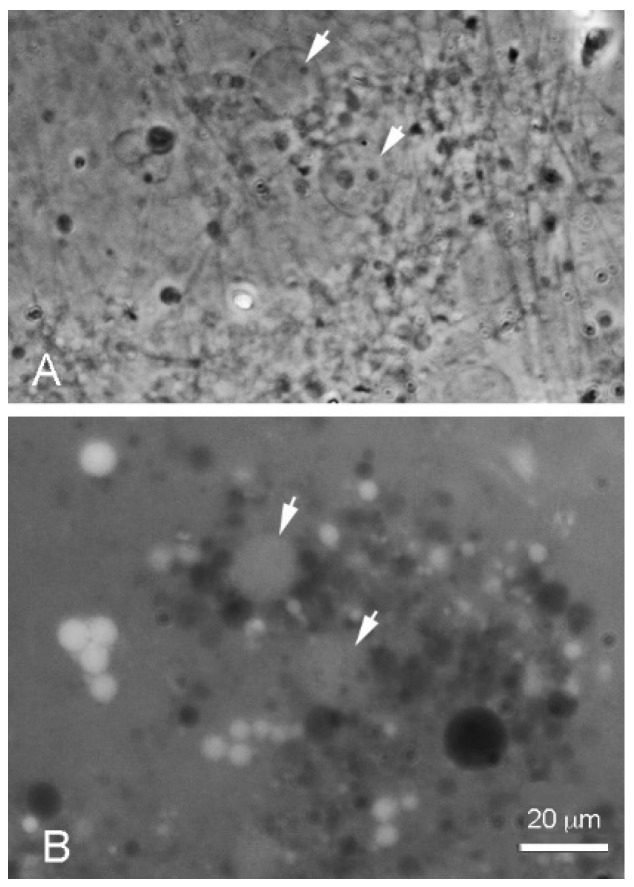
Dodecanoic acid (12 carbons) were mixed in a 1:1 mole ratio with its monoglyceride in pH 7.9 Bison Pool water, then were put through a wet-dry-wet cycle in the presence of 0.1 mM pyranine dye. Large vesicles are barely visible in the phase image (arrows, A) because the very thin bilayer membranes are not clearly resolved. However, the encapsulated pyranine was revealed within vesicles by fluorescence microscopy (B). Some vesicles have excluded the dye for reasons explained in the text.

Because the mixture of dodecanoic acid with its monoglyceride formed stable vesicles, we decided to determine whether the membranes offered a permeability barrier to solutes. Therefore, we repeated the experiment illustrated in Figure 4, but with 0.1 mM pyranine dye present as a fluorescent marker. Pyranine (8-hydroxy-1,3,6-pyrenetrisulfonate) has three negatively charged sulfonate groups that prevent it from readily permeating lipid bilayers. Figure 5 shows that when the LA–GML mixture was cycled in pH 7.9 Bison Pool water, large vesicles formed upon rehydration that encapsulated varying amounts of the dye. It is interesting that some vesicles did not contain dye and appeared black against the background fluorescence of free dye. This is typical of encapsulation by a wet–dry cycle, because when vesicles fuse into multilamellar structures some solutes are captured between the layers that formed when external surfaces of the original vesicles fused, while other solutes are excluded from the internal volumes of the fusing vesicles [14].

In order to establish how well the membranes could maintain solute gradients, LA–GML vesicles were put through a Sephadex G-50 gel permeation column after being exposed to a wet-dry-wet cycle in the presence of pyranine. The vesicles appearing in the excluded volume clearly contained dye and could be visualized by fluorescence microscopy. The amount of dye was determined from its excitation–emission spectrum taken by an Eclipse-Cary spectrofluorimeter and represented approximately 6% of the original dye present in the mixture.

### 3.4. Encapsulation of Nucleic Acids

It was also important to determine whether the LA–GML mixture can encapsulate nucleic acids. Therefore, the LA–GML mixture in pH 3.3 Geyser Basin water was dried in the presence of Torula yeast ribosomal RNA in a 2:1 ratio by weight, and also with lambda DNA, which has 48,000 base pairs. Acridine orange dye (0.1 mM) was added as a fluorescent stain.

**Figure 6:**
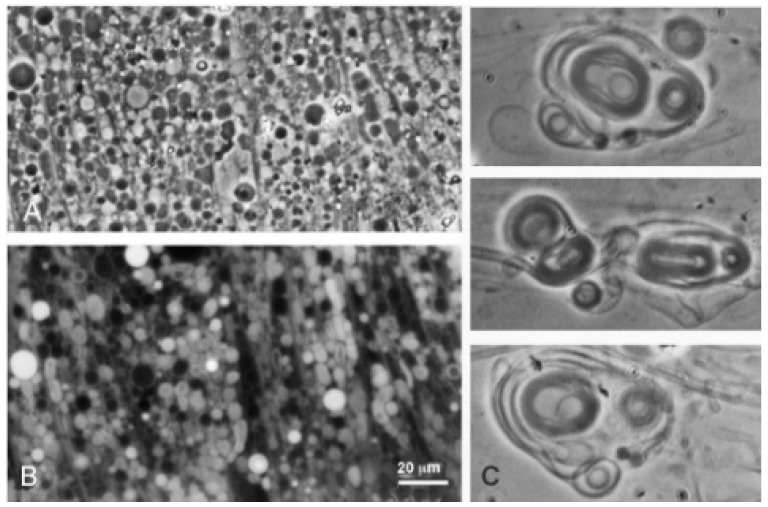
Lauric acid–glycerol monolaurate (LA–GML, 10 mM) was mixed with yeast ribosomal RNA in a 2:1 ratio by weight in pH 3.3 Geyser Basin water, then dried on a microscope and rehydrated with the same volume of water. Phase (**A**) and fluorescence (**B**) images. (**C**) A control in which the same lipid mixture was prepared without a wet–dry cycle and put through the gel permeation column in the absence of RNA.

Figure 6A,B shows phase and fluorescence micrographs of the result with RNA, and it is clear from the fluorescent stain that RNA has been encapsulated in some of the vesicles. Figure 6C shows a control in which the lipid mixture was prepared in the absence of RNA and put through the gel permeation column. The vesicles are much larger, multilamellar and more heterogeneous than the cycled sample.

A NanoDrop instrument was used to determine how much RNA was encapsulated, and in two repeats of the experiment was found to be 35% and 38% of the original amount present.

**Figure 7:**
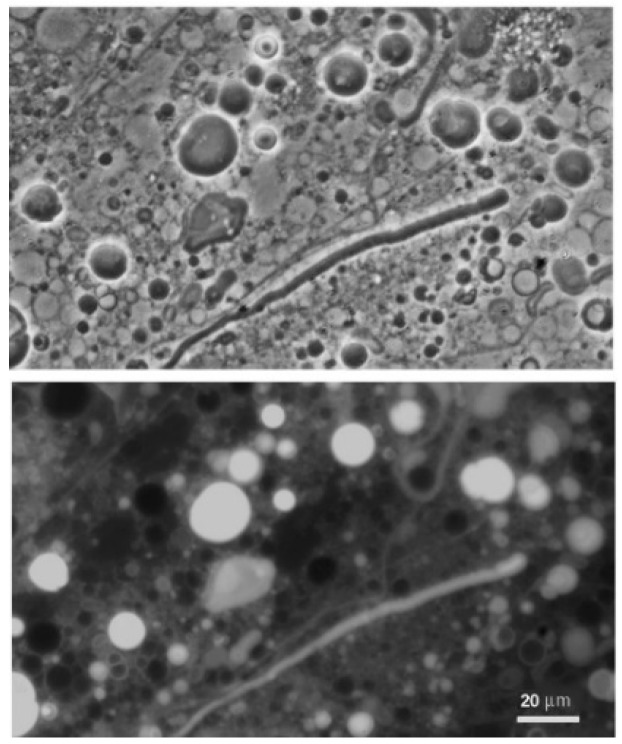
LA–GML (10 mM) was mixed with lambda DNA in a 2:1 ratio by weight in pH 3.3 Geyser Basin water, then dried on a microscope slide and rehydrated with the same volume of water. Phase (**top**) and fluorescence (**bottom**) images.

Figure 7 shows the same experiment with lambda DNA, and the encapsulated DNA is again clearly apparent from the fluorescent stain. The vesicles containing DNA tended to be larger in diameter (2–10 µm) compared to those containing RNA (2–5 µm).

### 3.5. Seawater Strongly Inhibits Assembly of Membranes

**Figure 8:**
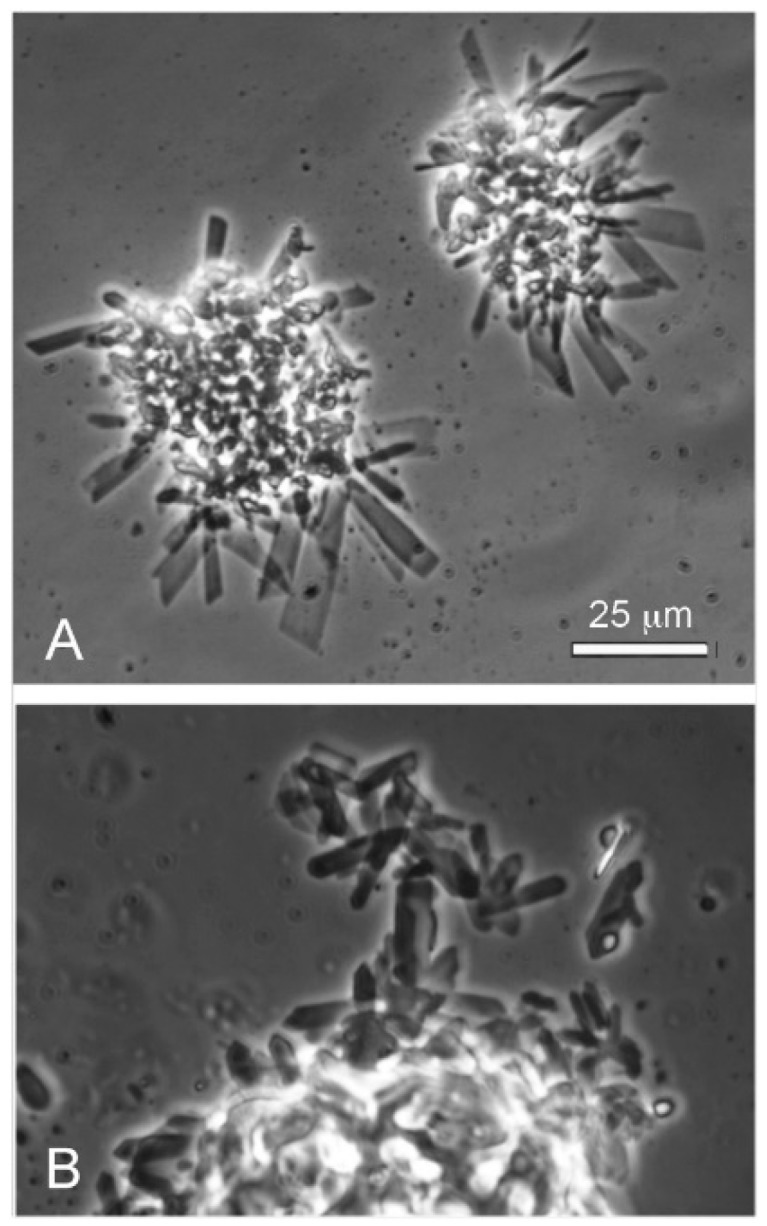
Dodecanoic acid (12-carbon lauric acid) in seawater formed crystals protruding from a central mass (**A**). A mixture of dodecanoic acid and its monoglyceride also formed particulate aggregates surrounded by crystals (**B**).

We compared the effect of seawater on lauric acid by itself, and also the LA–GML mixture. Pure lauric acid in seawater formed crystals rather than vesicles (Figure 8A), presumably because seawater contains divalent calcium and magnesium cations (10 mM Ca^++^ and 54 m Mg^++^). At the alkaline pH range of seawater (pH 8.1), the divalent cations bind to the carboxylate groups and form insoluble soaps that cannot assemble into membranous vesicles. The LA–GML mixture of dodecanoic acid and its monoglyceride also formed particulate aggregates surrounded by crystals (Figure 8B).

**Figure 9:**
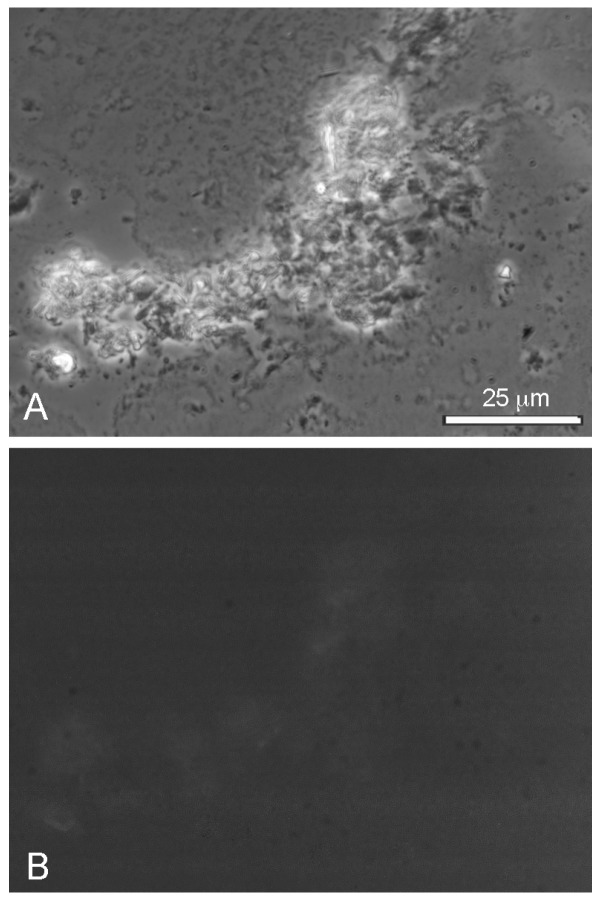
LA–GML put through a wet-dry-wet cycle in seawater formed intractable aggregates (**A**) that did not encapsulate pyranine dye (**B**).

Tide pools at shoreline go through multiple cycles of wetting and drying. For this reason, we also carried out a wet–dry cycle of the LA–GML mixture with pyranine dye present. Upon rehydration, no membranous structures were present. Instead, the amphiphiles formed large aggregated clumps (Figure 9A) lacking any trace of encapsulated dye (Figure 9B). The 10 mM Ca^++^ and 54 mM Mg^++^ in seawater became highly concentrated during drying and strongly interacted with the carboxylate groups on the lauric acid to form a hard water soap that was unable to assemble into membranes.

## 4. Discussion

The main conclusions from the results reported here is that, although pure decanoic and dodecanoic acid are able to form membranous vesicles in hydrothermal water samples from hydrothermal pools, they are relatively sensitive to temperature and pH. Similar results have been reported for self-assembly of amphiphilic fatty acid in an Indian geothermal environment [13]. In contrast, a mixture of a fatty acid and its monoglyceride (LA–GML) assembles into stable membranous vesicles in hot spring water at alkaline and acidic pH ranges and is less sensitive to pH. Similar results have been reported earlier under laboratory conditions [9,10], so the results reported here confirm that the dilute ionic solutes of hydrothermal water are not a barrier to vesicle assembly in analogue prebiotic conditions. We also observed that, if exposed to a wet–dry cycle, the LA–GML vesicles encapsulated pyranine, a fluorescent dye, as well as RNA and DNA. In contrast, none of the amphiphilic compounds formed membranes in seawater, nor could encapsulation of dye molecules be observed. 

### 4.1. Composition and Concentration of Ionic Solutes in Hydrothermal and Seawater

**Figure 10:**
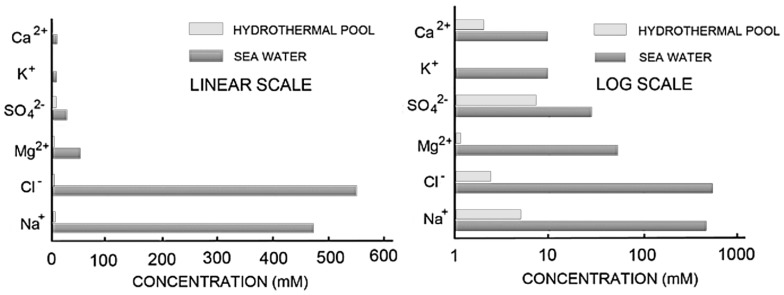
Ionic composition of seawater compared with hydrothermal water from Mammoth Hot Springs in Yellowstone National Park. Data from [16].

Before continuing the discussion further, it is important to understand the differences in ionic composition between hydrothermal water of hot springs and seawater. This is illustrated graphically in Figure 10, which clearly illustrates the large differences between the ionic concentrations of seawater and Yellowstone water, particularly for NaCa^+^, Ca^++^ and Mg^++^. This difference is important for two reasons. First, the half molar (~1.0 osmolar) concentration of NaCl in seawater produces an osmotic pressure gradient across the membranes of amphiphiles, which inhibits their ability to expand into membranous vesicles. The high concentration of divalent cations in seawater (54 mM Mg^++^ and 10 mM Ca^++^) also inhibits assembly of amphiphilic compounds into membranes because they interact with carboxyl and phosphate groups and cause amphiphiles such as fatty acids to collapse into aggregates [9]. In earlier work, we reported that a 1:1 mixture of decanoic acid and decylamine can assemble into remarkably robust membranous vesicles at high and low pH ranges [15], and also in seawater. However, long-chain amines have never been reported in extracts of carbonaceous meteorites nor as Fischer–Tropsch products, so we do not consider alkyl amines to be plausible components of prebiotic mixtures of organic compounds.

Table 1 in the Results section reports concentrations of ionic solutes in the hydrothermal water samples used in this study, and Table 2 shows data from a previous survey of Yellowstone water for comparison.

There are several important points to make about these results. First, the ionic solute concentrations of hydrothermal water taken from different sources in Yellowstone hydrothermal fields vary from micromolar to 27 mM total concentration of cations and anions (Table 2), and our results reported in Table 1 fall within this range. Second, the ionic solutes of seawater (Table 3) far exceed the highest concentrations of hydrothermal water. Such high concentrations inhibit membrane assembly and should be taken into account by proposals that life could begin in a marine environment. It is important to note that when dilute ionic solutes are exposed to a wet–dry cycle, they become highly concentrated in the dry phase. For instance, if the Mammoth Hot Spring water undergoes drying, the 27 mM ionic solutes pass through a concentration equivalent to ~2 molar when 99% of the water has evaporated. This must also be considered by proposals that wet–dry cycles in hydrothermal fields can drive polymerization and encapsulate products [17,18,19].

During review of this manuscript, a concern was expressed that perhaps unidentified organic substances or even microorganisms might contribute to the microscopic images, and that the hydrothermal water should have been filtered as we did with seawater. We do not share this concern. The hydrothermal water is remarkably pure, with a dissolved organic carbon content of just 49 and 44 micromoles per liter (see Table 1). Furthermore, it has been virtually sterilized by the near boiling temperatures at the hot spring source, and the 10 mM fatty acid mixtures dominate the microscopic fields. We decided it was best to simply add the hot water to the fatty acid mixtures directly from the source, then reheat the samples as needed during the six months in which they were later studied.

### 4.2. Effect of pH and Temperature on Fatty Acid Membranes

It has been known since the 1970s that fatty acids, such as oleic acid, can form stable membranous vesicles [20,21], and this fact has later been used to model prebiotic membranes. However, it is also understood that pure fatty acids can only assemble into membranes at a pH near the pK of the carboxylate groups, where hydrogen bonds forming between –COOH and –COO– stabilize a bilayer structure [22]. The hydrothermal Yellowstone water samples ranged in pH from acidic (pH ~3.3) to slightly alkaline (pH ~7.9), so it was essential to find mixtures of amphiphiles that were stable over a similar pH range. As will be discussed below, mixtures of fatty acids with their monoglycerides markedly improved stability to pH variations.

It was surprising that pure fatty acids could apparently assemble into typical vesicles and myelin figures in the acidic hydrothermal water (Figure 2 and Figure 3). In previous laboratory simulations, protonated fatty acids are present only as oil droplets [9]. An explanation for this effect is still uncertain, but it is possible that low concentrations of other ionic solutes in the hydrothermal hot spring water tended to interact with the head groups in such a way that bilayer structures were stabilized. Perhaps for the same reason, decanoic acid formed membranous structures even though its concentration of 10 mM was below the reported CVC (critical vesicle concentration) determined from laboratory preparations in which micellar dispersions were titrated to the pK at which membranes assembled.

Fatty acid vesicles can only assemble into stable membranous vesicles if the aqueous phase is at a temperature above the gel–fluid transition temperature of the amphiphilic compounds. The temperatures of the hydrothermal pools from which water samples were taken were well above the melting points of decanoic acid (31 °C) and dodecanoic acid (43 °C), but the fatty acids were in the gel phase at room temperature. For this reason, it was necessary to heat the samples, as described in Methods, and microscopy was performed while the slide was still warm. If the slide was allowed to cool, the vesicles collapsed into crystalline structures when the temperature fell below the melting points of the fatty acids.

### 4.3. Effect of Monoglycerides in Stabilizing Membrane Structure

Mixtures of fatty acids with glycerol esters can assemble into vesicles in hydrothermal water at both alkaline and acid pH ranges. The effect of lower temperature was less apparent in the mixtures of lauric acid with its monoglyceride. Although fluidity was reduced below the transition temperature, LA–GML remained as vesicles rather than collapsing into semi-crystalline structures as the pure fatty acid would. In prebiotic conditions, amphiphilic compounds would have been present not as pure species, but instead in a mixture of other solutes that could affect stability. For instance, the stabilizing effect of monoglycerides may have been amplified by the presence of certain bases and sugars that inhibit NaCl-induced precipitation of fatty acids [23]. However, the high salt and divalent cation concentrations in seawater would be likely to disrupt self-assembly of both fatty acid vesicles and their mixtures with monoglycerides whether or not other solutes were present.

### 4.4. Encapsulation of Nucleic Acids

Previous studies have demonstrated that DNA [22] and RNA [24,25,26] can be encapsulated by amphiphilic vesicles composed of fatty acid mixtures with monoglycerides. The results reported here extend these observations to confirm that encapsulation of nucleic acids in stable membranous vesicles can also occur in water samples from a hydrothermal field. The amount of RNA that was encapsulated (~35%) might be surprising to researchers who prepare liposomes in solutions of nucleic acids in which the ratio of internal to external volume might be 1:100, so that only 1% of the solute is encapsulated. However, the wet-dry-wet encapsulation method described here is much more efficient because the lipid vesicles fuse into a multilamellar structure during drying. The solutes, including polymers, become highly concentrated between lipid bilayers. Upon rehydration, the multilamellar structures swell and bud off into lipid vesicles that have the concentrated solutes remaining inside. Therefore, the ratio of internal to external volume is not the determining factor.

An interesting observation was that vesicles in the absence of RNA that were not exposed to a wet-dry-wet cycle were much larger than those cycled in the presence of RNA (Figure 6A–C). This suggests that the presence of an encapsulated polymer has a stabilizing effect and led to a more homogeneous, smaller population of vesicles after cycling.

## 5. Conclusions

We can now answer the questions posed in the introduction.

How does the ionic composition of hydrothermal hot spring water compare with seawater?

The most important properties of ionic solutes related to membrane stability are osmotic effects and binding of divalent cations to anionic head groups of amphiphiles. Ionic solutes in seawater are ~100-fold higher in osmotic concentration than in hydrothermal water, and 10- to 100-fold higher in divalent cation content.

Can model amphiphilic compounds assemble into stable membranes in hot spring water?

Monocarboxylic acids alone or mixed with monoglycerides form vesicles in hydrothermal water and may even be stabilized by certain dilute ionic solutes. The results confirm earlier reports that monocarboxylic acids with chain lengths of 10 or more carbons can assemble into membranous vesicles in laboratory conditions. Addition of monoglycerides markedly stabilized the vesicles over a range of pH and temperature.

Can the model amphiphilic compounds form vesicles in seawater?

The concentrated ionic solutes of seawater completely inhibit assembly of monocarboxylic acids into membranous vesicles either with or without admixture of their monoglycerides.

Can membranes assembled from model amphiphilic compounds provide permeability barriers to ionic solutes?

The LA–GML mixture represents a significant permeability barrier to an ionic solute. Pyranine dye stays in the vesicles long enough (~10 min) so that 6% remained after they are passed through a Sephadex G-50 column.

Can nucleic acids be encapsulated in vesicles assembled from model amphiphilic compounds?

With the LA–GML mixture in hydrothermal water samples, a single wet-dry-wet cycle captured RNA and DNA in vesicles. The nucleic acids could be observed in the vesicles by their fluorescence when stained with acridine orange. The encapsulated RNA represented over 30% of the original amount present in the mixture and was protected against hydrolysis by RNase.

## Figures and Tables

**Table 1:** Ionic composition of Yellowstone hot spring water used in the present study. Concentrations are shown as millimolar (mM) units.

**‘Bison Pool’ Outflow, Lower Geyser Basin**	
Temperature	68.4 °C
pH	7.9
Na	12.52 mM
Ca	0.01 mM
Mg	0.08 µM
K	0.36 mM
DIC (HCO_3_^−^)	5.42 mM
Cl	6.12 mM
F	1.21 mM
SO_4_	0.17 mM
SiO_2_ (dissolved)	3.60 mM
Cationic equivalents	12.90 mM
Anionic equivalents	13.09 mM
DOC (dissolved organic carbon) 49.4 micromoles per liter	
**Midway Geyser Basin**	
Temperature 53.0 °C	
pH	3.3
Na	0.39 mM
Ca	0.02 mM
Mg	0.01 mM
K	0.17 mM
DIC (H_2_CO_3_)	0.17 mM
Cl	0.14 mM
F	0.03 mM
SO_4_	0.32 mM
SiO_2_ (dissolved)	(not measured)
Cationic equivalents	1.12 mM
Anionic equivalents	0.67 mM
DOC (dissolved organic carbon) 43.8 micromoles per liter	

**Table 2:** Ionic composition of Yellowstone hot spring water [16]. Concentrations are shown both as ppm and as millimolar (mM).

**Mammoth Hot Springs T =**	**71.5 ± 2 °C**	**(N = 7)**	**Mean**
pH	6.5 ± 0.3	(N = 6)	6.5
Na	131 ± 7 ppm	(N = 8)	5.7 mM
Cl	166 ± 3 ppm	(N = 9)	4.6 mM
Ca	305 ± 60 ppm	(N = 6)	7.6 mM
Mg	68 ± 5.7 ppm	(N = 7)	2.8 mM
K	55 ± 8 ppm	(N = 7)	1.4 mM
HCO_3_	725 ± 135 ppm	(N = 5)	12 mM
SO_4_	510 ± 38 ppm	(N = 7)	5.2 mM
SiO_2_	53 ± 3.8 ppm	(N = 8)	0.9 mM
Cationic equivalents	27.9 mM		
Anionic equivalents	28.2 mM		
**Norris Geyser Basin T =**	**86 ± 6 Degrees °C**	**(N = 20)**	**Mean**
pH	4.1 ± 0.7	(N = 14)	4.1
Na	299 ± 130 ppm	(N = 18)	13 mM
Cl	611 ± 171 ppm	(N = 10)	17 mM
Ca	5.9 ± 3.8 ppm	(N = 16)	0.150 mM
Mg	0.6 ± 0.3 ppm	(N = 16)	0.026 mM
K	53 ± 26 ppm	(N = 38)	1.4 mM
H_2_CO_3_	~0		
SO_4_	131 ± 68 ppm	(N = 4)	1.3 mM
SiO_2_	450 ± 123 ppm	(N = 9)	7.5 mM
Cationic equivalents	14.7 mM		
Anionic equivalents	18.3 mM		

**Table 3:** Ionic composition of seawater. Concentrations are shown as millimolar (mM).

Sea Water	pH = 8.1
Na	469 mM
Ca	10 mM
Mg	53 mM
K	10 mM
HCO_3_	2 mM
SO_4_	28 mM
SiO_2_	0
Cationic equivalents	605 mM
Anionic equivalents	604 mM
